# Nonlinear Model-Based Inferential Control of Moisture Content of Spray Dried Coconut Milk

**DOI:** 10.3390/foods9091177

**Published:** 2020-08-26

**Authors:** Zalizawati Abdullah, Farah Saleena Taip, Siti Mazlina Mustapa Kamal, Ribhan Zafira Abdul Rahman

**Affiliations:** 1 Department of Process and Food Engineering, Faculty of Engineering, Universiti Putra Malaysia, Serdang 43400, Malaysia; zalizawati8653@uitm.edu.my (Z.A.); smazlina@upm.edu.my (S.M.M.K.); 2 Faculty of Chemical Engineering, Universiti Teknologi MARA, Shah Alam 40450, Malaysia; 3 Department of Electrical and Electronic Engineering, Faculty of Engineering, Universiti Putra Malaysia, Serdang 43400, Malaysia; ribhan@upm.edu.my

**Keywords:** inferential control, spray drying, one-dimensional, NARX, neural network, moisture content, coconut milk powder

## Abstract

The moisture content of a powder is a parameter crucial to be controlled in order to produce stable products with a long shelf life. Inferential control is the best solution to control the moisture content due to difficulty in measuring this variable online. In this study, fundamental and empirical approaches were used in designing the nonlinear model-based inferential control of moisture content of coconut milk powder that was produced from co-current spray dryer. A one-dimensional model with integration of reaction engineering approach (REA) model was used to represent the dynamic of the spray drying process. The empirical approach, i.e., nonlinear autoregressive with exogenous input (NARX) and neural network, was used to allow fast and accurate prediction of output response in inferential control. Minimal offset (<0.0003 kg/kg) of the responses at various set points indicate high accuracy of the neural network estimator. The nonlinear model-based inferential control was able to provide stable control response at wider process operating conditions and acceptable disturbance rejection. Nevertheless, the performance of the controller depends on the tuning rules used.

## 1. Introduction

Spray drying process is one of the most widely used methods to convert liquid to powder in food processing. Spray dryer is commonly used in production of powder products such as milk powder [1,2,3], fruit and vegetable powder [4,5,6], and encapsulated flavor [7,8,9]. In spray drying process, the liquid is converted to powder via rapid evaporation. The liquid is atomized by an atomizer to form small droplets and mixes with hot medium. The numerous small droplets have a large surface area that allows the fast removal of moisture by evaporation process while maintaining its spherical shape. The particles (dehydrated droplets) fall through the gaseous medium to the bottom of drying chamber and collected in dryer collector [10]. The dried product is the best solution for easier transportation and storage with higher microbiological stability compared to in liquid phase [11]. Moisture content of powder is one of the most important parameters to be maintained as it indicates product quality and also influences other product properties, especially flow properties [12]. Low moisture content is desirable as its related to low water activity of powder, which is important for powder stability during storage [13]. The moisture content of spray dried products must be lower than 5% with water activity of less than 0.6. For high fat content powder, water activity around 0.3 is most stable against lipid oxidation and microbial deterioration [14]. Dried powder needs to be stored properly as water activity of powder might change over time due to absorption of water, which affected by temperature and relative humidity of storage area [15].

In industry, moisture content cannot be measured in real time due to low reliability of measuring device as well as consideration of hygienic issues of the food product. Due to this factor, outlet drying temperature has been widely selected as controlled variable as it is directly correlating to the final moisture content of products [16]. Proportional-integral-derivative (PID) control is widely used to control the outlet temperature due to easy implementation, easy tuning, and having good control performance [17]. Even though PID control is still valid to be used, its application to control the outlet air temperature to retain the powder quality is unsatisfactory. This is due to fact that the relationship between outlet air temperature to moisture content might vary due to changes in other variables such as relative humidity [18] that leads to changes in controller performance [19].

Application of advanced process control to moisture content of powder is highly promising but challenging. In advanced process control, the presence of an accurate dynamic model in designing the controller is highly important. Several approaches can be used to represent the dynamic of the spray drying process, i.e., course, finer, and finest models [20]. A finer model also known as one-dimensional model consists of mass, heat, and momentum balance of individual droplets within the overall mass and energy balances [21]. This model is capable of predicting the average air flow and droplets behavior, especially the temperature, velocity, and moisture content, at different drying durations and dryer heights, relatively in a short period of time [22]. This capability makes it reliable to be used in moisture content control system, but a highly accurate model can only be obtained by multiscale model integrating the drying kinetic model in the one-dimensional model in order to capture the dynamic behavior of specific material under consideration.

Recently, the reaction engineering approach (REA) model has been widely used as a drying kinetic model of food in particulate and thin layer form. Patel and Chen [23] integrated the REA model in one-dimensional model and it was found out that this model is simpler in its mathematical formulation and superior in providing realistic results, compared to other lumped parameter drying kinetic models to represent the spray drying process of skim milk. George, et al. [24] discovered that a one-dimensional model with integration of REA model provides a good fit to experimental data that can accurately predict the dynamic behavior of the co-current spray dryer.

The application of the dynamic model, especially a one-dimensional model, allows the application of an advanced control system in controlling moisture content of spray dried product such as cascade control [25], linear–quadratic–Gaussian (LQG) controller [26], and model predictive control [27]. However, the dynamic model is computationally intensive for real time applications due to time consuming issues in iteration of a complex model [24]. A simplified version of the nonlinear dynamic model is required to reduce simulation time in online control strategy such as linearization [28] and reduction [26] of a nonlinear model. However, Govaerts et al. [26] found out that the reduced model failed to provide good close-loop control response. On the other hand, a linear model tends to be insufficient in controlling the process in a wider range of operating conditions. Instead of one-dimensional model, empirical formulation is well-known to be able to provide nonlinear relationship of the process with a fast output response. Neural network is the most widely used formulation to predict the quality of spray dried powder [29]. However, this data-driven model requires more experimental work to allow prediction over a wider operating range on the process [30]. Furthermore, the time-series data of moisture content cannot be obtained as only the residual moisture content can be assessed from the spray drying process.

From the industrial point of view, PID-based advanced control system such as inferential control is more cost-effective compared to other types of advanced control system [31]. Due to this fact, this study is conducted by leveraging on the advantages of both fundamental model and empirical models in development of advanced control system to control the moisture content. The objective of this research is to develop a nonlinear model-based inferential control to control the moisture content of spray dried coconut milk. In this study, the dynamic model is developed by integrating the REA model in the one-dimensional model to represent the dynamic in spray drying process. The developed one-dimensional model is simplified using an empirical approach. The empirical models, i.e., nonlinear autoregressive with exogenous input (NARX) model and neural network (NN) model are used in model-based inferential control as system identification (process model) and soft sensor estimator, respectively.

## 2. Materials and Methods

### 2.1. Dynamic Model of the Spray Drying Process

#### 2.1.1. Drying Kinetic Study

The drying kinetic of coconut milk has been studied using single droplet drying, i.e., sessile droplets drying in convective drying tunnel [32]. The drying kinetic model is formulated based on reaction engineering approach (REA) model.

#### 2.1.2. Experimental Setup of Drying Tunnel

An aluminum tunnel with dimension of 5 × 12 cm (height × width) is customized to create a small convective drying tunnel. Axial flow fan created laminar flow in the tunnel, which was fed from one end of the drying tunnel. The air is heated using a rod heater and the temperature is controlled at 80 ± 3 °C A low humidity condition in the tunnel was created using silica gel, which also serves as a porous medium. The relative humidity was maintained at 10% while velocity of air is maintained at 0.3 m/s. The air velocity and relative humidity were measured using Testo 425 hot wire anemometer and D3121 Thermo-hygrometer (Comet System), respectively. Measurements are to be taken prior to sample positioning in the drying tunnel. Under controlled environment, the single droplet drying can mimic convective drying of droplet in spray drying chamber [33].

**Figure 1:**
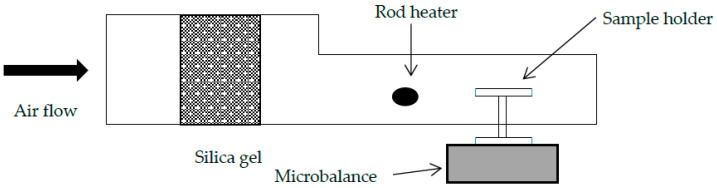
Drying tunnel for single droplet drying.

The sample holder was placed on a microbalance to provide continuous measurement of droplet’s weight. The drying tunnel and sample holder were equilibrated at temperature 80 °C for 1 h before the experiment was conducted. The drying tunnel is shown in Figure 1.

#### 2.1.3. Sessile Droplet Drying

In this study, Teflon sheet was used as hydrophobic surface in sessile droplet drying. Teflon sheet was attached on the 0.4 mm aluminum sheet to form sample plate with size 8.5 × 20 mm. One droplet of 5 μL was placed on sample plate by using micropipette. The plate was then placed on sample holder, and weight loss was measured continuously throughout the drying process. The change in droplet size was continuously recorded by a video camera. The droplet temperature was measured at the center of the droplet using thermocouple in separate run and was assumed uniform throughout the droplet during the drying process.

#### 2.1.4. Development of Drying Kinetic Model

The REA model is used to describe the drying kinetic of coconut milk. To formulate this model, several parameters need to be determined from experimental work. The most important parameter requiring measurement is mass decrease of droplets, which represents removal of moisture from emulsion through drying process. The gradient temperature of droplet during drying process also needs to be measured. One of the most accurate drying curves was selected to be used in drying rate, dX/dt derivation.

#### 2.1.5. Mathematical Formulation of Reaction Engineering Approach Model

Chen and Xie [34] formulated a reaction engineering approach (REA) model where the model expresses drying rate as shown in Equation (1):

(1) $$\frac{{dm}_{p}}{dt}=m_{s}\frac{dX}{dt}=-h_{m}A_{p}\left[\exp\left(-\frac{\Delta E_{v}}{R_{G}T_{s}}\right)\rho_{v,s}\left(T_{s}\right)-\rho_{v,b}\right]$$

where $m_{p}$ and $m_{s}$ are the mass of product (kg) and mass of dry matter (kg), respectively. X is the average moisture content (kg/kg, db), dX/dt is the water evaporation rate (kg/ (kg·s), $h_{m}$ is the mass-transfer coefficient (m/s), $A_{p}$ is the product surface (m^2^), $\Delta E_{v}$ is the apparent activation energy, $R_{G}$ is gas constant (J/mol·K), and $T_{s}$ is the droplet interface temperature. $\rho_{v,s}$ and $\rho_{v,b}$ are the water vapor concentration at droplet interface and in drying air (kg/m^3^), respectively. Calculation of apparent activation energy is done based on rearrangement of Equation (1). The derivative dX/dt is determined from the drying curve, i.e., moisture content over time.

Mass-transfer coefficient ($h_{m})$ is calculated using Sherwood correlations, which relates the sample geometry and flow conditions in the drying tunnel. In this study, Sherwood number (Sh) from Ranz-Marshall correlation (Equation (2)) for sessile droplet was used [32].

(2) $$Sh=(h_{m}.d_{s})/D_{v}=0.24+0.62{Re}^{0.51}\cdot {Sc}^{1/3}$$

In this correlation, $d_{s}$ is the sphere diameter (m), $D_{v}$ is the vapour-air diffusivity (m^2^/s), Re is Reynolds number, and Sc is Schmidt number [35]. Activation energy can be linked to the moisture content of the sample as formulated in Equation (3):

(3) $$\Delta E_{v}/\Delta E_{v,b}=f\left(X-X_{b}\right)$$

where $\Delta E_{v,b}$ is the equilibrium activation energy, i.e., the maximum activation energy at drying temperature ($T_{b})$. The $\Delta E_{v,b}$ is calculated using Equation (4):

(4) $$\Delta E_{v,b}=R_{G}T_{b}\ln(\rho_{v,b}/\rho_{v,sat}(T_{b}))$$

The relationship between the relative activation energy to moisture content can be correlated using simple nonlinear regression equation. $X_{b}$ is the equilibrium moisture content, i.e., the moisture content at which the material is neither gaining nor losing moisture. Equilibrium moisture content is determined at corresponding drying temperature and relative humidity. This study utilized hot air drying to determine equilibrium moisture content of coconut milk [36] at similar drying temperature and relative humidity of the convective drying. The equilibrium moisture content is determined when no further mass variation of sample can be observed, whereas initial moisture content of the sample is determined from hot air oven. The oven temperature is maintained at 105 °C and the sample are to be dried for 3 h.

To validate the developed model, the comparison of the moisture content from the simulation of REA model with experimental data was carried out. The comparison has been made by observing changes in moisture content of sample dried at 90 °C within a certain period. In this study, coefficient of determination, *R*^2^ value was used throughout this paper to determine the fitness of the time-series model for the given data set. Equation (5) is used to calculate the *R*^2^:

(5) $$R^{2}=1-\frac{\sum_{i=1}^{N}{\left(Y_{i}^{exp}-Y_{i}^{pred}\right)}^{2}}{\sum_{i=1}^{N}{\left(Y_{i}^{exp}-{\bar{Y}}_{i}^{pred}\right)}^{2}}$$

where $Y_{i}^{exp}$ and $Y_{i}^{pred}$ are the experimental outputs and model output, respectively. ${\bar{Y}}_{ave}^{exp}$ corresponds to average value of experimental data. The analysis was conducted in a Microsoft Excel spreadsheet.

### 2.2. Mathematical Formulation of One-Dimensional Spray Dryer Model

One-dimensional model based on the unsteady state mass and energy balances adapted from George et al. [9] is used to describe the dynamic of spray drying process. The model is expressed based on the assumption that spray drying behaves as a co-current plug flow reactor and droplets have spherical shape, which bears identical properties at the same altitude in drying chamber. Several other assumptions of the spray drying process made to simplify the mathematical formulation are listed as follows:

- The air behavior is close to that of an ideal gas, therefore the properties of air can be determined based on ideal gas law
- Hold-ups of dry air and solid powder are constant; therefore, the flowrate of dry powder is equal for stream entering and leaving the chamber
- The pressure in the dryer remained at atmospheric pressure

The moisture content of the powder is determined based on its drying kinetics, Equation (1). The change in bulk air humidity is based on the mass transfer of water from droplet to the air (Equation (6)):

(6) $$\frac{dY}{dt}=\frac{{\theta m}_{s}\frac{dX}{dt}}{F_{p}}$$

The energy balance is used to describe the outlet temperature profile of the droplet temperature (Equation (7)) and hot gas (Equation (8)):

(7) $$\frac{{dT}_{p}}{dt}=-\frac{\left[h_{h}A_{p}\left(T_{b}-T_{p}\right)-m_{s}\lambda \frac{dX}{dt}\right]}{m_{w}C_{p,water}+m_{s}C_{p,solid}}$$

(8) $$\frac{{dT}_{b}}{dt}\;=\frac{1}{GC_{p,b}}\left[-\theta h_{h}A_{p}\left(T_{b}-T_{p}\right)+m_{s}\theta \frac{dX}{dt}C_{p,v}(T_{b}-T_{p})-Q_{loss}\right]$$

where $m_{w}$ is mass of water (kg) and $m_{s}$ is mass of powder (kg), $F_{p}$, *G*, $T_{p}$ and $T_{b}$ are flowrate of droplet (kgs^−1^), flowrate of air (kgs^−1^), droplet temperature (K), and hot air temperature (K), respectively. Y is absolute humidity of air (kg water kg dry air^−1^), λ is the latent heat of water vaporization (Jkg^−1^), $h_{h}$ is the heat transfer coefficient (Js^−1^m^−2^K^−1^), θ is number of particle size and $A_{p}$ is the area of the droplet (m^2^). $C_{p,water}$, $C_{p,solid},$ and $C_{p,b}$ are the specific heat of water, dried particle, and air (Jkg^−1^K^−1^), respectively.

Convective heat transfer coefficient, $h_{h}$ is calculated using Ranz-Marshall correlation for forced around spherical bodies and can be calculated by Equation (9) [22]:

(9) $$Nu=(h_{h}.d_{s})/k_{b}=2+0.6{Re}^{1/2}\cdot {Pr}^{1/3}$$

In this correlation, $d_{s}$ represents sphere diameter (m) and Re represents Reynolds number. Heat loss to the surrounding from the dryer is calculated by Equation (10) [37]:

(10) $$Q_{loss}=U_{p}\pi DL(T_{b}-T_{amb})$$

where $U_{p}$ is the heat loss coefficient (Wm^−2^K^−1^), *D* is diameter of chamber (m), *L* is length of chamber (m), and $T_{amb}$ is ambient temperature (K). Other related equations are listed in Appendix A.

The mass and energy balance are solved with fourth order Runge-Kutta method in conjunction of several algebraic equations. The subroutine of the model was developed in the Matlab^®^ environment with built-in ODE45 Runge-Kutta method. Variables and properties used in the model are listed in Table 1. Data and values are obtained from experimental work, assumption, and previous studies. The heat capacity of solid is obtained from the differential scanning calorimetry (DSC) analysis of the coconut milk powder.

Table 2 presents inlet conditions of the spray dryer where all the inlet conditions for the model development is based on the spray drying process conducted in this research. The moisture content of the coconut milk was obtained from oven drying [38]. The coconut milk fed to the spray dryer consists of coconut milk emulsion homogenized with 2% sodium caseinate and 8% of maltodextrin. Water was added to the grated coconut meat with weight ratio of 1:1 prior to extraction of the emulsion. The simulation was carried out using a time step of 1 s.

In this study, the equilibrium moisture content (EMC) is formulated based on assumptions that relative humidity (RH) of the spray drying process is below 40% [41] and the moisture content is 0% at 0% of RH. Study of EMC for whole milk by Lin, et al. [41] observed that at low RH, EMC is independent of drying temperature and possess linear correlation with RH. The relation between EMC and relative humidity is represented in Equation (11):

(11) $$\mathrm{EMC}=\alpha (\rho_{v,b}/\rho_{v,sat}(T_{b}))$$

Model validation is conducted by comparing outlet temperature and residual moisture content from simulation of one-dimensional model with experimental data. The experimental work has been achieved by spray drying coconut milk emulsion in co-current lab-scale spray dyer (Labplant SD 05). Validation of model is conducted by varying inlet temperature, i.e., 140, 160, and 180 °C, while other variables are fixed at values listed in Table 2.

### 2.3. Formulation of Empirical Models

#### 2.3.1. NARX Model Development

One-dimensional model is used to generate dynamic time-series data to be used in system identification. In this study, nonlinear autoregressive with exogenous input (NARX) formulation is used in nonlinear system identification. Inlet temperature of the one-dimensional model has been varied from 120 to 200 °C in order to capture wider operating range of spray drying process. Simulated time-series data consists of 826 sets of data that were used to formulate the model, and the modelling process was conducted using System Identification Toolbox in MATLAB environment.

Two dynamic NARX models are developed to represent the process model in controller design. These models were used to correlate inlet temperature with outlet temperature, and inlet temperature with moisture content. Both models are formulated based on the general model shown in Equation (12):

(12) $$y\left(t\right)=f\left(\begin{array}{c} y\left(t-1\right),y\left(t-2\right),\ldots .,y\left(k-n\right),u\left(t-1\right),\\ u\left(t-2\right),\ldots .,u\left(t-n\right)) \end{array}\right)$$

where the output y(t) is regressed from the past value of output, y and past value of input, *u*. In this equation, *f* is the nonlinear estimator. The selection of the model parameter, i.e., number of regressors was determined based on the best fit data. The developed NARX models are saved in MATLAB Workspace and they are imported to Simulink environment to represent the process model of the control system. The simulation of the models is conducted to observe the dynamic profiles produced from the developed models.

#### 2.3.2. Neural Network (NN) Estimator Development

In an inferential control system, the nonlinear estimator is proposed to the feedback control to estimate the moisture content due to changes in the inlet temperature and the outlet temperature. Neural network was used in this research as it has been successfully used as a soft sensor (estimator) of moisture content of dried milk [42]. It is also due to fact that neural network is able to handle highly nonlinear correlation with multiple input output mapping. Simulated time-series data generated from the one-dimensional model were used in development of NN estimator as dynamic-based estimator to provide better control performance compared to static- and steady-state-based estimator [43]. Sets of data numbering 826 were fed to the neural network as the training, testing, and validation data. Seventy percent of the data was used as the training data. Another 15% was used for testing and 15% for validation. The designing of neural network estimator has been conducted in Matlab^®^ by using Neural Network Toolbox. Neural network with one input layer, one hidden layer, and one output layer was used as network architecture, and inlet temperature, and outlet temperature were selected as the input variables. The output layer consisted of one neuron that is the moisture content of the powder.

Two different types of nonlinear time series neural networks are used in development of NN estimator. For the first model, the input of the NN consists of past values of input variables and output variables also known as NARX. This scheme creates feedback connection to the network. Meanwhile, in the second NN model, the input of the NN consists of past values of input variables only, also known as nonlinear input-output. These models are named as NN-NARX and NN-N, respectively. The network was trained by Levenberg-Marquardt algorithm. Sigmoid activation functions were used for the hidden layer and output layer as it creates nonlinearity to the network. In this study, 10 nodes were fixed in hidden layer to avoid overfitting and underfitting of the developed model.

Mean squared error (MSE) was used as performance indicators of the network. The MSE is the error performance function, which is calculated based on value of average squared error between network outputs, $Y_{i}^{pred}$ and target outputs, $Y_{i}^{exp}$ as formulated in Equation (13):

(13) $$\mathrm{MSE}=\frac{1}{n}\overset{n}{\underset{i-1}{\sum }}{\left(Y_{i}^{pred}-Y_{i}^{exp}\right)}^{2}$$

### 2.4. Inferential Controller Design

**Figure 2:**
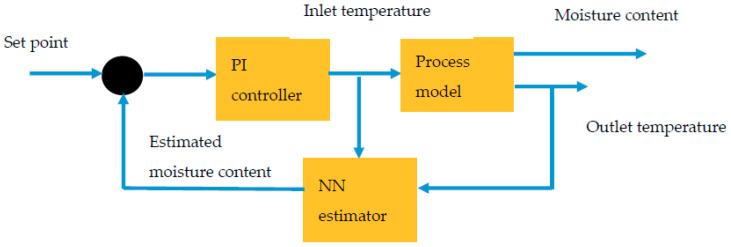
Inferential control loop scheme. PI, proportional-integral; NN, neural network.

The NARX models and NN model developed as discussed in previous section are integrated in MATLAB Simulink to design nonlinear inferential control system. NARX models and NN model represented as process model and estimator in the control system, respectively. Simulation of the inferential control is conducted in MATLAB Simulink software, and a block diagram of the inferential control is shown in Figure 2.

Based on the control loop scheme, inlet temperature is selected as the manipulated variable while controlled variable, i.e., moisture content can be predicted by measuring outlet temperature. The measured temperature is fed to neural network estimator and moisture content is predicted. The remaining of the process is similar to feedback control system. The system is considered as inferential control system because the moisture content measurement is not gained directly, but it is measured by using measurements of outlet temperature and inlet temperature. The linear proportional-integral (PI) controller is used as the control system. Algorithm of the controller is as in Equation (14):

(14) $$u\left(t\right)=Kc\left(e(t\right)+\frac{1}{\tau_{i}}\overset{t}{\underset{0}{\int }}e(t)dt)$$

Where Kc and $\tau_{i}$ are the controller parameters, i.e., controller gain and integral time, respectively. *u* is the controller output. Errors between the process value and set point value is notated as e.

Closed loop tuning rules have been used to determine the controller parameters. In this study, 3 different tuning rules have been chosen namely, Ziegler-Nichols (ZN), Tyreus-Luyben (TL), and Relaxed-Ziegler Nichols (R-ZN) [44]. The equations of the tuning rules are tabulated in Table 3. The controller parameters are initially determined by obtaining the ultimate gain, K_cu_ and ultimate period, P_u_ from trial-and-error of proportional-only controller. The controller performance is evaluated based on percent overshoot, setting time, and rise time.

### 2.5. Controller Performance

The controller performance is evaluated based on the capability of the controller to control the process due to set point change and presence of disturbance. The set point change has been conducted by making changes to the set point to high and low moisture content values. For disturbance rejection, ±15% change in inlet temperature is made and considered as disturbance to the control system.

Controller performance index is conducted using quantitative analysis, i.e., integral absolute error (IAE) and integral time absolute error (ITAE), which calculated from Equations (15) and (16), respectively:

(15) IAE=∫|e(t)|dt 

(16) ITAE=∫t|e(t)|dt

In these correlations, e(t) is error between the responses with the set point and t is time.

## 3. Results

### 3.1. Drying Kinetic Model of Coconut Milk Droplet

In formulation of REA model as the drying kinetic model, experimental work needs to be conducted to determine the activation energy of the evaporation process. Sessile droplet drying is to be conducted first, to determine drying rate, dX/dt which is derived from drying curve (weight loss of droplet versus time) of coconut milk droplet when heated at 80 °C.

**Figure 3:**
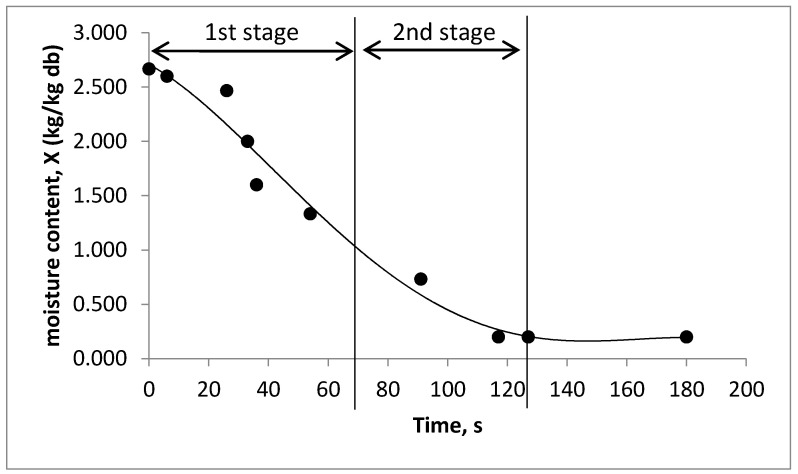
Drying curve of single droplet drying of coconut milk.

Figure 3 shows the drying curve obtained from convective drying of a single droplet of coconut milk. Similar trend of moisture loss was observed for droplet drying of encapsulated walnut oil [45], lactose, reconstituted skim milk, and reconstituted whole milk [46]. This indicates that a droplet undergoes the same drying nature regardless of droplet composition. Two main stages of drying can be differentiated from the drying curve. The 1st drying stage, i.e., constant rate period is observed for the first 70 s of drying process, which obtained from the linear part of drying curve. The 2nd stage, i.e., falling rate period occurs at 70 to 127 s, where the drying rate gradually decreases and reaches equilibrium moisture content. A longer 1st stage drying period is observed due to higher fat content of droplet where the hydrophobic surface hinders water vapor from evaporate [45]. Therefore, coconut milk droplets required a longer drying period to reach to equilibrium moisture content at medium drying temperature. Higher feed temperature or higher air temperature can be considered in spray drying process to reduce the constant drying rate period. However, too high temperature might lead to instability to the feed emulsion and dried powder.

**Figure 4:**
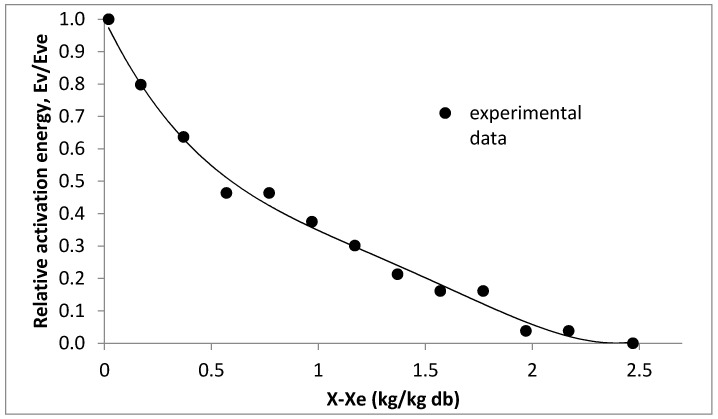
Reaction engineering approach (REA) approach fitted to normalized relative activation energy versus moisture content curve.

From the drying curve, drying rate, dX/dt is obtained, and is used to determine the activation energy as formulated in Equation (1). The average temperature and average surface area of the droplet are 50 °C and 1.42 × 10^−5^ m^2^, respectively. Large surface area of droplet and high droplet temperature allow water to evaporate faster as more area is exposed to hot air and water has more energy to escape from the droplet. Normalized relative activation energy, $\left(\Delta E_{v}/\Delta E_{v,b}\right)$ is calculated and plotted against difference between moisture content to equilibrium moisture content $\left(X-X_{b}\right),$ as shown in Figure 4. It can be seen from the graph that minimum energy is required when the droplet has high moisture content. Energy required to remove the water increases as less water is present in the droplet. The relative activation energy correlating to moisture content represented by 4th order polynomial equation (Equation (17)) with REA model fitted with the experimental data with *R*^2^ value of 0.9786. *R*^2^ > 0.8 indicates good fit of regression equation to observed data [47].

(17) $$\Delta E_{v}/\Delta E_{v,e}=0.1073{\left(X-X_{e}\right)}^{4}-0.6089{\left(X-X_{e}\right)}^{3}+1.2171{\left(X-X_{e}\right)}^{2}-1.1668\left(X-X_{e}\right)+1$$

Accurate relative activation energy leads to an accurate REA model, which can be used to predict global drying rate of coconut milk droplets at different drying conditions in spray drying chamber [48]. The global drying rate controlled the rate of moisture removal from droplets.

**Figure 5:**
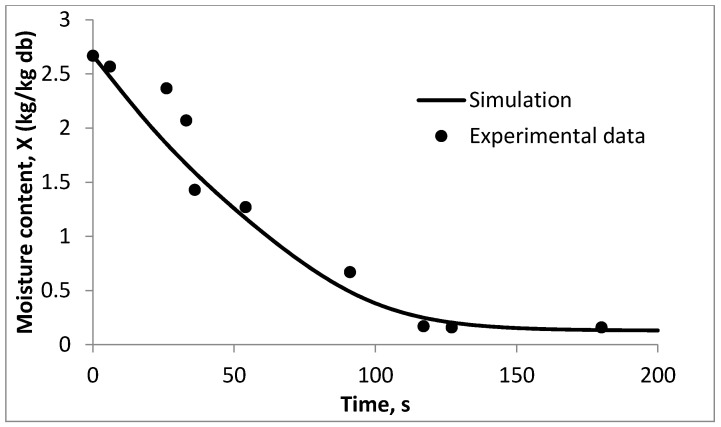
Validation of REA model with the experimental data with drying temperature of 90 °C.

Figure 5 shows the comparison of the model with validation data obtained for droplet drying at 90 °C. Results show that the profile of the model is in good agreement with validation data with the *R*^2^ value of 0.882. The developed kinetic model can be integrated to the one-dimensional model of the spray drying process.

### 3.2. One-Dimensional Model

**Figure 6:**
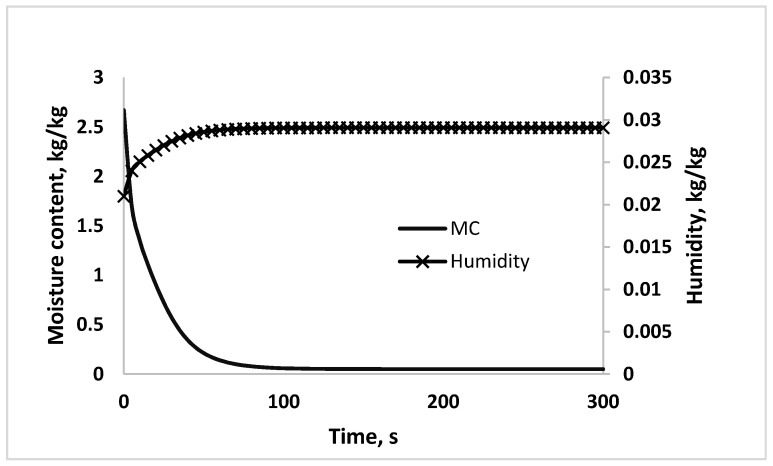
Simulated response of droplet moisture content and air humidity.

The REA model was integrated in the mass and energy balance of the co-current spray drying process. Figure 6 shows the dynamic profile of moisture content of the powder and humidity of hot air predicted by the one-dimensional model with inlet temperature fixed at 160 °C. The predicted dynamic profiles are obtained from Equations (1) and (6). Shorter constant drying period (<50 s) is observed to the simulated moisture content compared to droplet dried at 80 °C during single droplet drying. This is due to higher inlet temperature and smaller size of droplet used in simulation of spray drying process, which increase the mass transfer rate. An opposite trend is seen for air humidity profile, where the air humidity increased as the moisture content decreased. This is expected as water removal from droplet is being transferred to hot air.

**Figure 7:**
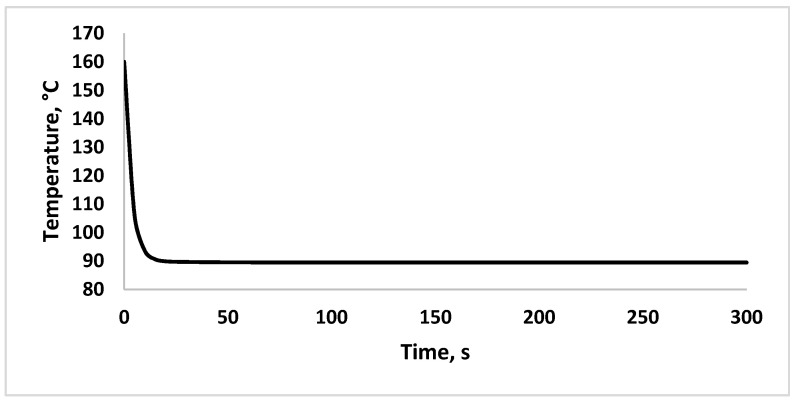
Simulated response of outlet temperature of hot air.

The simulated outlet air temperature profile is shown in Figure 7, which is obtained from Equation (8). As some heat is transferred to evaporate the droplets, the outlet air temperature decreases with the increase of time and finally reaches a new steady state. A similar finding was obtained by Palencia et al. [49] for dynamic modelling of outlet air temperature of co-current spray dryer. They found out that consideration of simultaneous heat and mass transfer between phases is crucial in modelling the dynamic of spray drying process for automatic control application.

**Figure 8:**
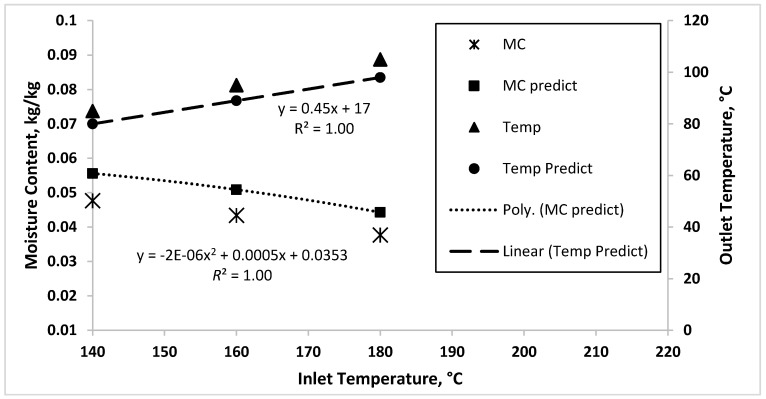
Comparison of experimental data of moisture content (MC) and outlet temperature (Temp) with simulation data.

Figure 8 illustrates the comparison between the simulated result and experimental data. The simulated results are slightly deviated from the experimental data. A possible cause for the deviation is that some parameters were obtained from literature, which might not represent the real process parameters. However, the simulated results have similar trends to the experimental data where the moisture content decreases as the inlet temperature increases, while the outlet temperature increases as the inlet temperature increases.

Linear increment of outlet temperature was observed with the increase of inlet temperature with perfect fit with linear regression with *R*^2^ of 1.00. This result is in good agreement with the relation of inlet and outlet temperature of co-current spray dryer studied by George et al. [9]. Slightly nonlinear relation is observed between inlet temperature and moisture content as the data perfectly fit the second order polynomial regression with *R*^2^ = 1.00. A similar trend is observed for moisture content obtained from experimental work.

To check the agreement between predicted values with the experimental results, the mean absolute percentage error (MAPE) was found to be 17.1% for moisture content and 6.2% for outlet temperature. These results indicates good prediction of the one-dimensional model as MAPE results between 10 to 20% show that the model has a good prediction capability [50]. The one-dimensional model provides accurate estimation for the relationship between variables within the spray drying process, indicating that the model is able to capture the dynamics of the process. On the other hand, the moisture content possesses nonlinear relationship with the inlet temperature and outlet temperature of hot air. Linearization of the process during process control design might affect the controller performance over a wide moisture content range.

### 3.3. NARX Model

The one-dimensional model is simulated with varied inlet temperature from 120 to 200 °C to generate dynamic data for model and estimator development. From the simulation results, the range of simulated outlet temperature and moisture content is 70.7–108.1 °C and 0.0367–0.0609 kg/kg, respectively. Two NARX models were developed from the dynamic data, namely NLARX1 and NLARX2. NLARX1 relates inlet temperature (T_in_) of hot air to outlet temperature of hot air (T_out_), while NLARX2 relates inlet temperature of hot air (T_in_) to moisture content of powder (MC). The wavelet network was used as nonlinearity estimator. In NARX model development, number of regressors, i.e., the number of past observations samples, play the most important role in capturing the nonlinearity of the system. It was found out that the NARX with one input and output regressor is the most accurate model to represent the process. A similar finding was obtained by Ramesh et al. [51] in NARX model development of distillation column, where one output regressor is sufficient to capture the nonlinearity of the process as its provide linear relation with process nonlinearity. In this study, higher number of regressor is not discussed as it leads to increase in complexity of the model, which consequently reduces the model accuracy. Meanwhile, the input regressor is crucial to be considered as it provides direction to the output.

High correlation was obtained from these formulations between model and dynamic data with fit to working data of 99.29 and 99.37% for NARX1 and NARX2, respectively. Based on these results, NLARX1 and NARX2 is represented by Equations (18) and (19), respectively.

NARX1 = T_out_(t) = *f*(T_out_(t − 1),T_in_(t − 1)) (18)

NARX2 = MC(t) = *f*(MC(t − 1), T_in_(t − 1)) (19)

**Figure 9:**
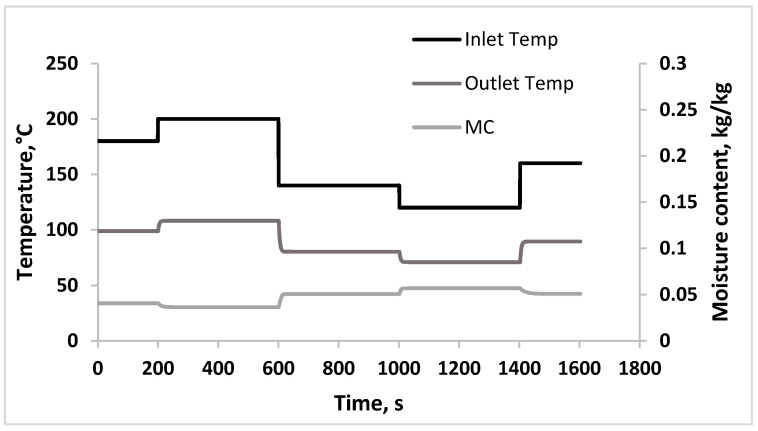
Simulated dynamic profile of outlet temperature (Outlet Temp) and moisture content (MC) of coconut milk powder subjected to multiple step change in the inlet temperature (Inlet Temp).

High accuracy of NARX models indicates that these models are able to represent the dynamic nonlinear of the drying process accurately. Simulated dynamic profiles obtained from NARX models for outlet temperature of hot air and moisture content of powder in function of time, when changes are made to the inlet air temperature is shown in Figure 9. As can be seen, the prediction of dynamical time-series data of process variables can be made at wide operating range of the spray drying process. The NARX models reduced the numerical complexity of process model and the prediction of the dynamic behavior is made in a short period of time. Therefore, these NARX models are feasible to be used as a process model in designing the inferential control system.

### 3.4. Neural Network Estimator

The selection of the NN estimator to be used in controller design is conducted by comparing the accuracy of the developed models in predicting the moisture content. The number of delays is set at 1 and 2 for both models. Table 4 shows the MSE value of NN-NARX and N-NN models during training and validation phases. It is found out that NN-NARX models are more accurate compared to NN-N models in predicting the moisture content of the powder with MSE value lower than 4.0 × 10^−9^ for training and validation phases. It is found out that neural network approach is able to provide high accuracy in predicting the dynamic nonlinear of the spray drying process regardless of network architecture used. This high accuracy is crucial to minimize the error between the estimated moisture content with its actual value as this error became the main contributor to static control offset in inferential control system [52]. It is also found out that NN-NARX models are more accurate compared to NN-N models in predicting the moisture content of the powder with MSE value lower than 4.0 × 10^−9^ for training and validation phases. It proves that previous output value provides useful insight of the system, therefore it provides better prediction of the model. A similar finding was obtained by Osman and Ramasamy [53] and Singh, et al. [54], where the NARX architecture outperformed other network architectures in soft sensor development using time-series data.

As both NN-NARX models are highly accurate to predict the moisture content of powder, the selection of the estimator is used based on the smallest MSE value of validation phase. Small error during validation phase compared to training phase indicates no overfitting condition as the model gives good prediction of the unseen data. Therefore, NN-NARX with number of delay of 1 is selected to be used as the estimator. The relationship between the input and output of the selected NN estimator is represented by the Equation (20):

MC(t) = *f*(MC(t − 1), T_out_(t − 1), T_in_(t − 1)) (20)

The developed neural network estimator was integrated to Simulink diagram and used in designing the inferential controller. The constant values from this network, i.e., weight and bias are used as the estimator to predict the moisture content of the powder.

### 3.5. Inferential Control of Moisture Content

In the food industry, the moisture content of the dried powder must be kept below 5% in order to extend the product shelf life. Therefore, in this study, moisture content of the powder will be varied close to this value and within the range of the process model. In this study, the linear proportional-integral (PI) controller was used as the controller. The optimum controller’s parameters were determined using three closed loop tuning rules, namely Ziegler-Nichols (ZN), Tyreus-Luyben (TL), and Relaxed-Ziegler Nichols (RZN) tuning rules. These tuning rules are selected as they are widely used to determine the controller parameters and their capabilities, especially in providing fast disturbance rejection and improved control stability [44].

The PI inferential controller parameters are listed in Table 5. Negative controller gains were obtained due to inverse response of the moisture content with the change of inlet temperature [42]. Based on the parameter values, ZN is expected to have fastest output response followed by R-ZN and TL. This is due to high value of Kc and small value of τ_I_, which leads to fast controller response and fast integral action, respectively.

**Figure 10:**
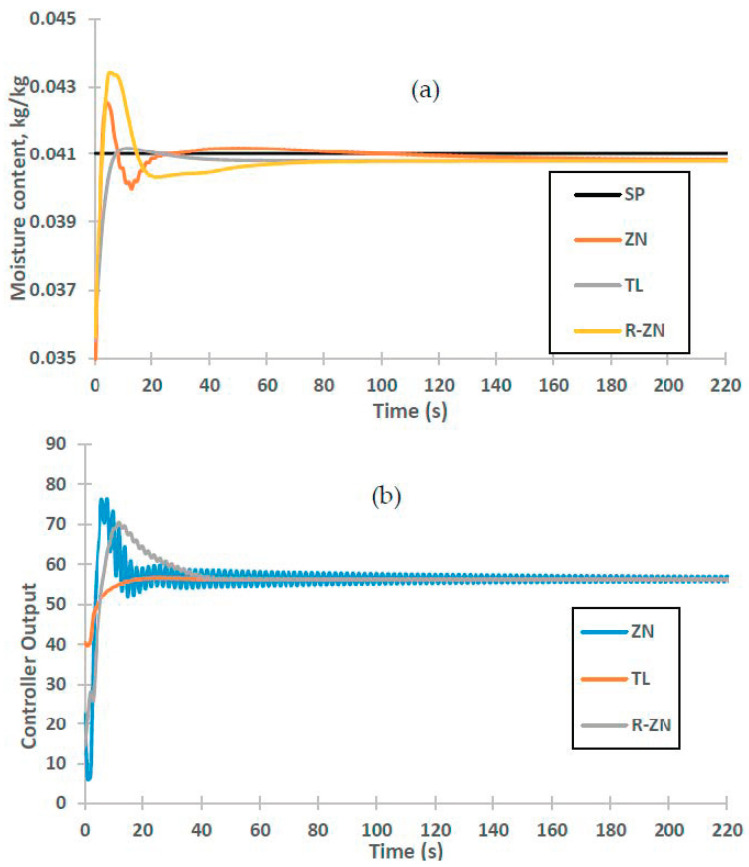
Response of inferential control system for Ziegler Nichols (ZN), Tyreus-Luyben (TL) and Relaxed-Ziegler Nichols (R-ZN) tuning rules. (**a**) Moisture content, (**b**) controller output.

Figure 10 shows moisture content of powder controlled using various tuning rules. It is observed in Figure 10a that all the PI inferential controllers are able to control the moisture content of the powder with minimal static control offset of less than 2.2 × 10^−4^ kg/kg. Static offset was observed by Kalbani and Zhang [52] during inferential composition control in distillation column and they found out that this offset can only be eliminated if true value of the variable is known.

Controller performance of the selected tuning rules is listed in Table 6. It is observed that TL-PI inferential controller has a good control performance as it produces minimum overshoot and faster setting time in the process response compared to other controllers. The overshoot and setting time values are 0.4% and 126 s, respectively. This might be due to smaller controller gain and larger integral time of this controller, which improves the controller output’s stability [44].

As can be seen in the controller output response in Figure 10b, TL-PI inferential controller produces stable controller output, which in turn produce good moisture content control response. Meanwhile, the other controller outputs almost reach to saturation limit at the beginning of the responses, which produce overshoot to the moisture content. As controller output is directly related to power of the air heater, high controller output leads to increase in power consumption.

### 3.6. Set Point Tracking

**Figure 11:**
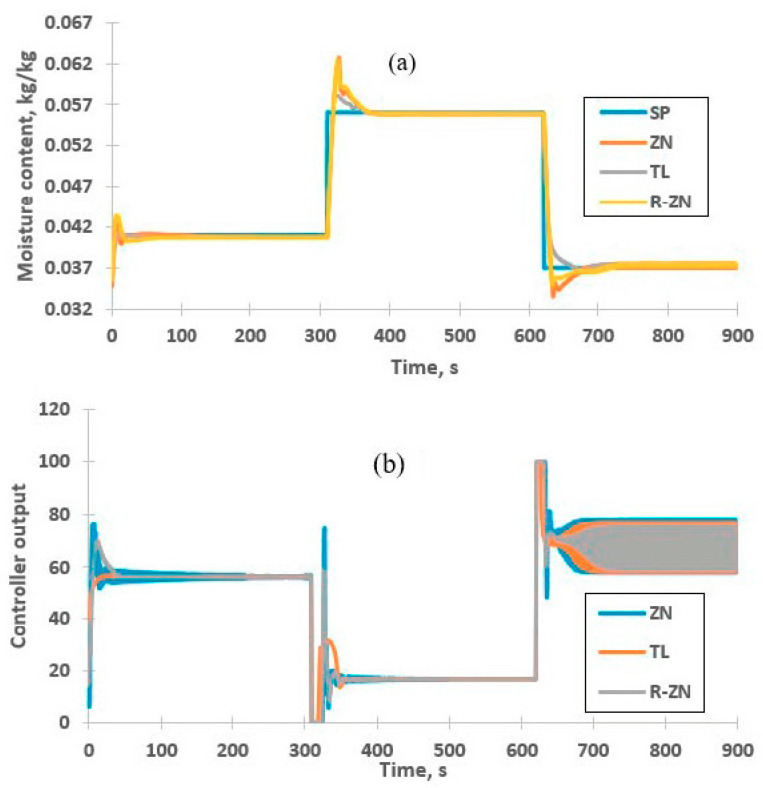
Response of inferential control system at various set points. (**a**) Moisture content, (**b**) controller output.

Result in Section 3.2 shows slightly nonlinear relation between inlet temperatures to moisture contents of powder. Changing the set point of nonlinear process might reduce the performance of linear PI controller. Therefore, changes were made to the set point to evaluate the performance of the controller. Figure 11 shows the powder moisture content when the step change is performed to the new set point, i.e., 0.056 and 0.037 kg/kg. It can be seen that all PI inferential controller settings are able to control the moisture content at high and low moisture content with minimal static control offset. The offset of responses at set point of 0.056 kg/kg is 0.58 × 10^−4^ kg/kg. Meanwhile, the offset of responses at low set point is less than 3 × 10^−4^ kg/kg. These results are tabulated in Table 7. This indicates the NN-NARX estimator has high accuracy to predict the moisture content of powder. TL-PI inferential controller outperforms other controller settings by producing a stable response with minimum overshoot at all set points. This is in agreement with error analysis, i.e., ITAE and IAE presented in Table 7. Based on these results, TL-PI inferential controller has the smallest error compared to other tuning rules at all set points with ITAE values less than 6.1 and IAE values less than 0.27. This indicates the good performance of the TL-PI inferential controller in controlling the moisture content of powder. It is also found that all responses create minimal offset with value less than 3 × 10^−4^ kg/kg.

Small oscillation is observed in all responses at set point of 0.037 kg/kg. This leads to high ITAE values for all PI inferential controller compared to at other set points. The high ITAE value indicates that the output response requires longer time to stable at its set point value as the ITAE value is calculated based on the integration of time with absolute error values. This is related to the sustained oscillation produced in controller output, as shown in Figure 11b. This is due to the linear nature of the PI controller, which slightly affects the controller performance at low set point. On the other hand, the IAE values is lower compared to response at set point of 0.056 kg/kg. These results reveal that the oscillations of these responses is smaller compared to oscillation produced at a high set point. The controller is still able to control the process at the set point value. Therefore, the inferential control is considered able to control the process at a wide operating range.

### 3.7. Disturbance Rejection

**Figure 12:**
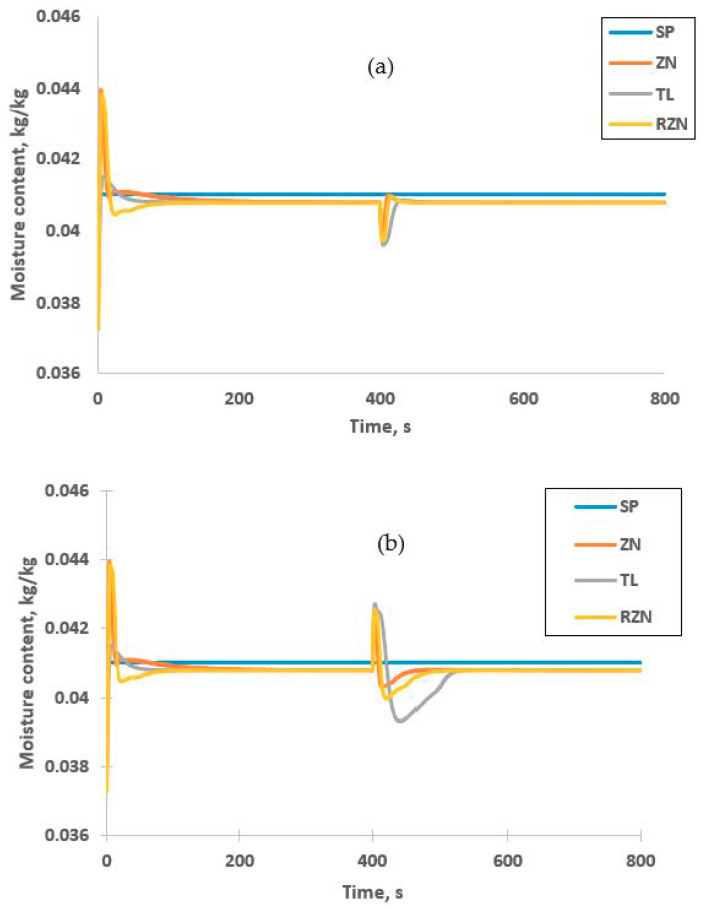
Response of inferential control system for disturbance rejection. (**a**) Positive deviation and (**b**) negative deviation.

The unmeasured disturbances can give negative impact to the controller’s performance as it leads to the deviation of response from its set point. Figure 12 shows the controllability of the PI inferential controllers when disturbance is introduced to the system. The unmeasured disturbance is set to cause ±15% deviation to the inlet temperature at 400 s after the process stabilized at set point. Figure 12a shows that all PI inferential controllers have a good performance in rejecting positive changes to the inlet temperature. Based on error analysis of the response tabulated in Table 8, all PI inferential controller have small error values with IAE less than 0.02 and ITAE less 0.4. These results reveal that all controllers provide good disturbance rejection with settling time less than 50 s.

When negative change is applied to inlet temperature at 400 s, ZN-PI inferential controller shows an excellent disturbance rejection, as can be seen in Figure 12b. Meanwhile, TL-PI inferential controller took longer time to reach to the set point value due to large overshoot. This resulted to large error value for both IAE and ITAE. This might be due to larger integral time of TL-PI inferential controller compared to other PI inferential controllers that lead to slower output response [55]. This is also due to high capability of ZN tuning rules in disturbance rejection [44]. Nevertheless, all responses are able to stabilize at set point, which indicates the capability of the PI inferential controllers to perform disturbance rejection.

## 4. Conclusions

From this study, it is found out that integration of REA model in one-dimensional model can accurately represent the dynamic behavior of spray drying process of coconut milk. This model allows the prediction of moisture content in function of time. Utilization of dynamic data from one-dimensional model allows the development of accurate NARX models and NN-NARX as system identification and inferential controller estimator, respectively. The developed NN-NARX estimator has high accuracy in predicting the moisture content based on the low static offset of responses at different set points. Result shows that TL-PI inferential controller shows good performance in set point tracking with small ITAE and IAE values, which indicates fast settling time and small overshoot of the process responses. On the other hand, ZN-PI inferential controller gave better performance in disturbance rejection. The proposed nonlinear based-based inferential control has a good control performance at a wider range of operating conditions and good disturbance rejection. It is revealed in this study that a fundamental model developed at multiscale, i.e., micro and macro scale, can be used to provide accurate prediction of dynamical time-series data of process variables and product qualities, which are known to be difficult to measure in food production. The presence of advanced computational tools allows fast prediction of complex nonlinear relationship of process variables, which are feasible to be used in design and improve and control of food processes in an attempt to produce high quality food products.

## Figures and Tables

**Table 1:** Variables and properties of spray drying process.

Variable/Property	Value	Reference
$C_{p,water}$	4185 J/kg·K	[39]
$C_{p,solid}$	1389 J/kg·K	Measured
$C_{p,v}$	1800 J/kg·K	[39]
$C_{p,b}$	1000 J/kg·K	[39]
$d_{s\;}$	200 μm	[24]
$v_{p}$	5 m/s	[40]
$v_{a}$	100 m/s	[40]
$R_{G}$	8.314 J/mol·K	[39]
λ	2501 × 10^3^ J kg^−^^1^	[39]
$T_{amb}$	300 K	Measured
$U_{p}$	22.8 W/m^2^·K	Calculated
L	0.5 m	Measured
D	0.22 m	Measured

**Table 2:** Inlet condition of spray dryer.

Variable	Value
Droplet:	
Moisture content	2.67 kg/kg
Temperature	27 °C
Flowrate	9.8 × 10^−4^ kg/s
Hot air:	
Humidity	0.021 kg/kg
Temperature	160 °C
Flowrate	0.011 kg/s

**Table 3:** Tuning rules of PI controller.

Tuning Rules	Controller Gain, Kc	$\mathrm{Integral}\;\mathrm{Time},\;\tau_{i}$
Zigler-Nichols (ZN)	0.45 K_cu_	P_u_ /1.2
Tyreus-Luyben (TL)	0.32 K_cu_	P_u_
Relaxed-Ziegler Nichols (R-ZN)	0.31 K_cu_	2.2 P_u_

**Table 4:** Mean squared error (MSE) values of neural network models.

Model	Number of Delay, d	MSE	
		Training	Validation
NN-NARX	1	3.960 × 10^−9^	8.678 × 10^−10^
	2	1.482 × 10^−9^	1.583 × 10^−9^
NN-N	1	7.589 × 10^−8^	1.327 × 10^−7^
	2	1.251 × 10^−7^	7.987 × 10^−7^

**Table 5:** PI inferential controller setting using various tuning rules.

Tuning Rules	Controller Gain, K_c_	Integral Time (s), τ_I_
Ziegler Nichols (ZN)	−12.75	1.67
Tyreus-Luyben (TL)	−8.79	4.40
Relaxed-Ziegler Nichols (R-ZN)	−9.072	2.00

**Table 6:** Controller performance using different tuning rules.

Tuning Rules	Overshoot (%)	Settling Time (s)	Rise Time (s)	Static Control Offset ×10^−4^ (kg/kg)
ZN	3.8	208	2	2.04
TL	0.4	126	11	2.18
R-ZN	5.9	100	2.9	2.18

**Table 7:** Integral absolute error (IAE), integral time absolute error (ITAE) and static control offset of inferential controllers for set point tracking.

Set Point (kg/kg)	Tuning Rules	ITAE	IAE	Static Control Offset ×10^−4^ (kg/kg)
0.041	ZN	2.0683	0.0461	2.04
	TL	0.1917	0.0205	2.18
	R-ZN	0.7400	0.0426	2.18
0.056	ZN	5.7465	0.3226	0.85
	TL	3.4722	0.2625	0.85
	R-ZN	5.7180	0.3260	0.85
0.037	ZN	7.8142	0.2081	0.48
	TL	6.0877	0.1531	2.00
	R-ZN	8.2237	0.2001	2.80

**Table 8:** Performance indices of inferential controllers for disturbance rejection.

Tuning Rules	Positive Deviation		Negative Deviation	
	ITAE	IAE	ITAE	IAE
ZN	0.237	0.008	0.761	0.023
TL	0.370	0.018	6.277	0.112
R-ZN	0.210	0.010	1.622	0.044

## Appendix A

### Correlations Used in REA Model and One-Dimensional Model Development

Water vapor concentrations in drying air $\left(\rho_{v,b}\right)$ and at droplet interface ($\rho_{v,sat})$ are calculated based on Equations (A1) and (A2), respectively [24].

(A1) $$\rho_{v,b}{=(P}_{sat}{.M}_{water}{)/\;(R}_{G}{.T}_{b})$$

(A2) $$\rho_{v,sat}{=(P}_{sat}{.M}_{water}{)/(R}_{G}{.T}_{p})$$

where $T_{b}$ is hot air temperature (K) and $T_{p}$ is droplet temperature (K), which is assumed equal to $T_{s}$. $P_{sat}$ is saturated vapor pressure and $M_{water}$ is molecular weight of water. $P_{sat}$ is calculated based on Clausius-Clapeyron relation as given in Equation (A3):

(A3) $${\;P}_{sat}\;=\left(\frac{101325}{760}\right)\exp\left(8.07131\;-\;\frac{1730.63}{T_{b}^{\prime }\;+\;233.426}\right)$$

where $T_{b}^{\prime }$ is bulk air temperature (°C) and $D_{v\;}$ is vapor-air diffusivity (m^2^/s), which is calculated from Equation (A.4). Reynolds number, Re, Schmidt number, Sc, and Prandtl number, Pr is calculated based on Equations (A5)–(A7), respectively [22].

(A4) $${\;D}_{v}\;=\;{0.22\times 10}^{-4}{\left(T_{b}/273.15\right)}^{1.75}$$

(A5) $${Re\;=\;(\rho }_{b}{|v}_{a}{-v}_{p}{\;|d}_{s}{)/\mu }_{b}$$

(A6) $${Sc\;=\;\mu }_{b}{/(\rho }_{b}D_{v})$$

(A7) $${Pr\;=\;(C}_{p,b}\mu_{b}{)/k}_{b}$$

where $T_{b}$ is hot air temperature (K), $v_{a}$ is velocity of air (m/s), $v_{p}$ is velocity of particle (m/s), $\mu_{b}$ is viscosity of air (kg/(ms)), $\rho_{b}$ is density of air (kg/m^3^), $C_{p,b}$ is specific heat of air (J/(kg.K)).
